# 30-day and 60-day rates and predictors of mortality among adult stroke patients: Prospective cohort study

**DOI:** 10.1016/j.amsu.2020.03.001

**Published:** 2020-03-21

**Authors:** Ginenus Fekadu, Legese Chelkeba, Tsegaye Melaku, Elsah Tegene, Ayantu Kebede

**Affiliations:** a Department of Pharmacy, Institute of Health Sciences, Wollega University, Nekemte, Ethiopia; b School of Pharmacy, Institute of Health, Jimma University, Jimma, Ethiopia; c School of Medicine, Institute of Medical Sciences, Jimma University, Jimma, Ethiopia; d Department of Epidemiology, Institute of Health, Jimma University, Jimma, Ethiopia

**Keywords:** Stroke, Predictor, Outcome, Mortality, Ethiopia

## Abstract

Stroke is one of the most common medical emergencies and the leading cause of preventable death and long-term disability worldwide. A prospective cohort study was conducted at the stroke unit of Jimma university medical center for four consecutive months (from March 10 to July 10, 2017). Of the total 116 study patients, 60 (51.7%) had an ischemic stroke. At 30-day follow-up, 81 (69.8%) patients were alive, 34 (29.3%) were died, and one patient (0.9%) was lost to follow-up. Elevated alanine aminotransferase (ALT) level (*AHR: 3.77, 95% CI: 1.34-10.57*), diagnosis of stroke clinically alone (*AHR: 3.90, 95 CI: 1.49-10.26),* brain edema (*AHR: 4.28, 95% CI: 1.61-11.37),* and National Institute of Health Stroke Scale (NIHSS) ≥ 13 during hospital arrival *(AHR: 6.49, 95% CI: 1.90-22.22)* were the independent predictors of 30-day mortality. At 60-day follow-up, 68 (58.6%) patients were alive, 46 (39.7%) were died, and 2 (1.7%) were lost to follow-up. Discharge against medical advice (*AHR: 6.40, 95% CI: 2.31-17.73)* and severe modified Rankin score/mRS (4–5) at discharge (*AHR: 3.64, 95% CI: 1.01-13.16)* were the independent predictors of 60-day mortality. The median (IQR) length of survival after hospital admission for patients died within 30 and 60 days were 4.65 (2.34–11.80) and 9.3 (3.93–33) days, respectively. Stroke significantly affects the morbidity and mortality in Ethiopia. There is a need to provide better care and future planning for stroke patients as an emergency diagnosis and treatment to minimize mortality and disability.

## Highlights

• Globally stroke is one of the most common medical emergencies and the leading cause of preventable death.
• More than 75% of stroke mortality is occur in low and middle-income countries.
• Stroke has become a major cause of morbidity, long-term disability and mortality in Ethiopia.
• In Ethiopia, resources for stroke care are deficient and majority of the patients are discharged with severe disability.
• The paucity of data specific to the Ethiopian setting limits the formulation of an appropriate intervention.

## 1 Introduction

Stroke is sudden brain cell death due to lack of oxygen when blood flow to the brain is lost by blockage or rupture of an artery in ≥24 h of symptoms onset, with no apparent cause other than vascular origin [[1], [2], [3], [4]]. It is one of the most common medical emergencies [5] and the leading cause of preventable death and long-term disability worldwide [[6], [7], [8], [9], [10], [11], [12]]. The burden of stroke is significantly high and is not only attributable to the high mortality and morbidity, but also to the high long-term disability [[13], [14], [15]]. It is also the leading cause of acquired disability and the third leading cause of mortality among women globally [16].

Stroke is an important disease globally putting pressure on the community health care system [17,18]. Additionally, it is a devastating condition associated with significant socio-economic costs [14,19,20]. The first-time incidence of stroke occurs in almost 17 million times a year worldwide and one in six people worldwide will have a stroke in their lifetime [18,21]. Early-onset mortality is common in hemorrhagic stroke (HS), whereas late mortality is prevalent among ischemic stroke (IS) [22].

Low and middle-income countries (LMICs) have the largest burden of stroke, consisting of more than 75% of stroke mortality globally and more than 80% of the total disability-adjusted life years [7,[23], [24], [25], [26]]. Globally, sub-Saharan Africa (SSA) has the highest incidence and case-fatality from stroke [10,27]. During the past several decades, the burden of stroke in the world has shifted from developed to developing countries [12,24]. This shift is thought to be driven by the aging of the population, population growth and changing patterns of diseases because of changes in risk factors and differences in socioeconomic status [12,28]. Unlike developing countries, stroke mortality is decreasing in the developed world [26,29]. The decreased percentage of stroke hospitalization and mortality in developed countries over the last decade likely reflect the advancements in acute stroke care [26,30,31].

The contribution of various risk factors to stroke globally is unknown, particularly in LMICs [7]. SSA is undergoing an epidemiological transition where stroke and other vascular diseases are increasingly [32]. The reasons for the high burden of stroke are linked to the high rates of risk factors like hypertension, diabetes mellitus, alcohol intake and smoking [33]. Various risk factors apply specifically to African community in the development of a stroke [34]. Additionally, the poor are increasingly affected because of both the changing exposures to risk factors and the inability to afford the cost of the medications [18,30,35]. Moreover, it remains uncertain if increased urbanization and life expectancy will shift the SSA region to a higher burden in the future [36].

In Ethiopia, stroke has become a major cause of morbidity, long-term disability and mortality [34]. The burden of IS and HS varies between the regions and over time [37]. Previous hospital-based studies conducted in Ethiopia reported that stroke patients suffer at a relatively young age and a higher proportion of HS than IS unlike Western countries [25,38]. The increasing burden of this stroke in Ethiopia poses a challenge to the health care system [39]. Resources for stroke care and rehabilitation are deficient [17,30]. Furthermore, in-hospital mortality is higher and the majority of the patients were discharged with severe disability [38]. This has a series of implications in terms of saving the life of patients which are characterized by a severe neurologic presentation [17].

Despite this, data on risk factors, clinical presentations, treatment outcomes and barriers to care of patients were scanty in Ethiopia among stroke patients [34,40]. This paucity of data in the country's setting limits the formulation of appropriate interventions [39]. The data for this study were part of a huge study project done in a stroke unit of Jimma university medical center with novel and extensive findings focusing on stroke. Therefore, the aim of the current study was to assess 30-day and 60-day mortality rates and predictors of mortality among adult patients admitted to the stroke unit of Jimma university medical center.

## 2 Patients and methods

### 2.1 Study setting, design, period and participants

The study was conducted at stroke unit of Jimma university medical center (JUMC), a tertiary hospital in Jimma city, south-west Ethiopia. It is the main referral center for neurology patients in southwest Ethiopia. A prospective cohort study was carried out for 4 consecutive months from March 10- July 10, 2017. Those patients died before evaluation, changed the initial diagnosis of stroke, patients diagnosed with a transient ischemic attack, hematomas, stroke transformation, undetermined type of stroke and readmitted cases were excluded from the study. One hundred sixteen (116) patients of ≥18 years of age full filling the inclusion criteria were included as described elsewhere [35,[41], [42], [43], [44]]. Hence, the characteristics of study participants in this finding share similarities with previously published articles of the same study project. The work has been reported in line with the strengthening the reporting of cohort studies in surgery (STROCSS) criteria [45].

### 2.2 Outcome and validating methods of measurements

Stroke case fatality (30 and 60-day mortality) was considered the primary outcome. Patients were followed from hospital arrival until the end of the study period. Within admission of 30 and 60 days mortality was assessed by close follow up of the patient through telephone interview of the patient/caregiver/proxy on a weekly basis after patients were discharged from the hospital. Since most patients were died in their homes without a death certificate, the death of patients was confirmed only by the information obtained from the patient's family/caregiver.

### 2.3 Data collection process

A pretest was done on 5% of patients to ensure the validity and reliability of the data collection tool. After pretesting, all necessary adjustments were made on the data collection instruments before implementing them in the main study. Data were collected by trained two nurses and one internal medicine resident using a checklist sheet from the medical records and interviews of the patients/caregivers. The needed history used for the study was taken from the patient and/or relatives in the language they understood. Initial neurological assessment was performed within 24 h of hospital arrival. The decision to perform different ancillary tests, laboratories, and imaging, as well as clinical history taking was left to the treating clinicians. Using the data collection tool, all relevant information about each patient such as sociodemographic characteristics, clinical information, treatment outcomes, length of survival and outcomes was recorded.

### 2.4 Data processing and analysis

Checked data was cleaned and entered into Epidata version 3.1 and analysis of data was carried out using the Statistical Program for Social Sciences (SPSS) version 20.0. Continuous variables were presented in mean (standard deviation) or median (inter-quartile ranges) when skewed in distribution. Categorical variables were presented as frequency and proportions. A chi-square test was used to test the association between stroke outcome and various categorical variables. Trends within 30-day and 60-day mortality after stroke were compared before and after adjustment for covariates. We computed the overall unadjusted weighted proportions of stroke hospitalizations that resulted in death across time. Because adequate significant variables were obtained at p < 0.05, it was considered as a cutoff point for candidate selection and those identified variables at p < 0.05 on binary cox regression were subjected to multivariable cox regression with a backward stepwise approach to identify predictors of mortality at 30 and 60 days. The 30 and 60-day mortality rate of different predictor variable were compared using Kaplan–Meier method and the significance of the difference were checked using the log-rank test. Statistical tests were considered significant when the p-value was <0.05. The data analysis and correlation were done based on the selected variables for addressing all specific objectives adequately. Finally, the results were assessed and presented by using charts, tables, and texts.

### 2.5 Ethics approval and consent to participate

Ethical clearance was obtained from the Institutional Review Board (IRB) of Jimma University, Institute of health with the reference number of IHRPGC/107/207. Permission was obtained from the responsible bodies of the JUMC and stroke unit prior to the interview and review of the patient data. At hospital, written informed consent was obtained from the study participants. All patients got the right to opt-out of the research. For patients that have altered levels of consciousness or severe aphasias, an appropriate substitute decision-maker (i.e. close family member, relatives/proxy/caregiver) was given the consent. Additionally, verbal consent was obtained from patients or a next-of-kin and the telephone numbers of those who accepted to participate were obtained. The data from the case records and the interview was handled with strong confidentiality. Neither the case records nor the data extracted were used for any other purpose. The confidentiality and privacy of patients were assured throughout by removing identifiers from data collection tools using different codes. The study was registered at researchregistry.com with a unique reference number of “researchregistry5260”.

## 3 Result

### 3.1 30-day baseline characteristics and follow-up outcome

Of the total 116 study patients, 60 (51.7%) patients had an IS while, 56 (48.3%) had HS with intracerebral hemorrhage (ICH) and subarachnoid hemorrhage (SAH) accounted for 44.0% and 4.3%, respectively. The mean age of patients who died within 30 days was 53.26 ± 14.23 years while those alive was 55.86 ± 14.05 years. Both male and female stroke patients died within 30 days were more common in rural than in the urban without a statistical difference (p = 0.547). From the sociodemographic and other baseline characteristics, none of them had any association with stroke outcome at 30-day upon the chi-square (p > 0.05) (Table 1).

**Table 1:** Sociodemographic and other baseline characteristics and 30-day mortality among adult patients admitted to stroke unit of JUMC from March 10-July 10, 2017.

Variables		Total (n = 116)	Died at 30-day (n = 34)	Alive at 30-day (n = 81)
Age (years)	Mean ± SD	55.14 ± 14.04	53.26 ± 14.23	55.86 ± 14.05
	<45	26 (22.4%)	11(32.4%)	15(18.5%)
	45–65	65 (56.0%)	16(47.1%)	48(59.3%)
	>65	25 (21.6%)	7(20.6%)	18(22.2%)
Sex	Male	73 (62.9%)	23(67.6%)	50(61.7%)
	Female	43 (37.1%)	11(32.4%)	31(38.3%)
Residence	Rural	84 (72.4%)	24(70.6%)	59(72.8%)
	Urban	32 (27.6%	10(29.4%)	22(27.2%)
Marital status	Married	104 (89.7%)	32(94.1%)	72(88.9%)
	Widow	11 (9.5%)	2(5.9%)	9(11.1%)
	Divorced	1(0.9%)	–	–
Ethnicity	Oromo	82 (70.7%)	30(88.2%)	51(63.0%)
	Kafa	15 (12.9%)	1(2.9%)	14(17.3%)
	Amhara	8 (6.9%)	1(2.9%)	7(8.6%)
	Dawuro	6 (5.2%)	2(5.9%)	4(4.9%)
	Others	5 (4.3%	–	5(6.2%)
Religion	Muslim	71 (61.2%)	26(76.5%)	44(54.3%)
	Orthodox	35(30.2%)	7(20.6%)	28(34.6%)
	Protestant	9 (7.8%)	1(2.9%)	8(9.9%)
	Traditional belief	1(0.9%)	–	1(1.2%)
Education status	Unable to read and write	42 (36.2%)	14(41.2%)	27(33.3%)
	Able to read and write, informal education	49 (42.2%)	12(35.3%)	37(45.7%)
	Elementary school (1–8)	17 (14.7%)	6(17.6%)	11(13.6%)
	Secondary school (9–12)	3 (2.6%)	1(2.9%)	2(2.5%)
	College/university or above	5 (4.3%)	1(2.9%)	4(4.9%)
Occupational status (over the last 1years)	Agriculture/farmer	44 (37.9%)	13(38.2%)	31(38.3%)
	Homemaker/housewives	41 (35.3%	12(35.3%)	28(34.6%)
	Merchant	11 (9.5%)	1(2.9%)	10(12.3%)
	Retired	6 (5.2%	2(5.9%)	4(4.9%)
	Government employee	5 (4.3%)	1(2.9%)	4(4.9%)
	Other own business work	5 (4.3%)	2(5.9%)	3(3.7%)
	Skilled/unskilled manual labor/daily worker	4 (3.4%)	3(8.8%)	1(1.2%)
Employment level	Unemployed- own/self-work	101 (87.1%)	28(82.4%)	72(88.9%)
	Unemployed – Retired	6 (5.2%)	2(5.9%)	4(4.9%)
	Professional employment	5 (4.3%	1(2.9%)	4(4.9%)
	Casual employment	4 (3.4%)	3(8.8%)	1(1.2%)
Body mass index (BMI) (kg/m^2^)	Mean ± SD	21.22 ± 3.38	20.94 ± 3.14	21.43 ± 3.40
	≤18.5 (underweight)	24 (20.7%	6(17.6%)	17(21.0%)
	18.6–24.9 (normal)	74 (63.8%)	25(73.5%)	49(60.5%)
	25.0–29.9 (overweight)	18 (15.5%)	3(8.8%)	15(18.5%)
Home distance of the patient from hospital (km)	Median (IQR)	45 (18–100)	45(18–68.03)	47.9(14.0–106.10)
	≤10 km	23 (19.8%)	5(14.7%)	18(22.2%)
	10.01–50 km	46 (39.7%)	17(50.0%)	28(34.6%)
	50.01–100 km	20 (17.2%)	8(23.5%)	12(14.8%)
	>100 km	27 (23.3%)	4(11.8%)	23(28.4%)
Living situation during pre-stroke	Independent at home	99 (85.3%)	29(76.5%)	70(86.4%)
	Dependent at home	14 (12.1%)	4(11.8%)	9(11.1%)
	hospital/health center	3 (2.6%)	1(2.9%)	2(2.5%)
Feeding habits	Mixed diet	95 (81.9%)	26(76.5%)	69(85.2%)
	Non vegetarian	15 (12.9%)	5(14.7%)	9(11.1%)
	Vegetarian	6 (5.2%)	3(8.8%)	3(3.7%)
Approximated monthly income (ETB)	Median (IQR)	500 (200–1000)	500(137.5–872.5)	500(200–1100)
	<500 birr	66 (56.9%)	22(64.7%)	43(53.1%)
	501-1000 birr	25 (21.6%)	7(20.6%)	18(22.2%)
	>1000 birr	25 (21.6%)	5(14.7%)	20(24.7%)

*Ethnicity others: Silte, Yem, Tigire, Nuwer; ETB: Ethiopian birr, SD: standard deviation; IQR: interquartile range.

At 30-day follow-up, 81 (69.8%) patients were alive, 34 (29.3%) patients were died, and one patient (0.9%) was lost to follow-up. From those patients that were alive for 30 days, 80 (98.8%) were present in their home and 1(1.2%) was in the health facility.

The median (IQR) length of survival after hospital admission for stroke patients who died within 30 days was 4.65 (2.34–11.80) days and the median length of survival after hospital discharge was 13.58 (8.99–17.40) days. The median (IQR) length of survival after hospital admission for ischemic and hemorrhagic stroke patients who died within 30 days was 4.75 (2.25–19.67) days and 4.58 (2.35–10.32) days, respectively. The median (IQR) length of survival after hospital discharge for ischemic and hemorrhagic stroke patients who died within 30 days was 12.75(8.28–18.77) days and 13.75 (8.48–17.40) days, respectively.

The 30-day crude mortality was 23 (41.1%) for hemorrhages and 11 (18.3%) for IS which was statistically significant (HR: 2.56, 95% CI: 1.25–5.25, p = 0.011). The 30-day case fatality was lower in the rural community (28.6 vs. 31.2%), but it was higher in males (31.5% vs. 25.6%). There was a significant difference between patients who were died and survived to 30 days from hospital admission with respect to NIHSS (National Institutes of Health Stroke Scale) and GCS (Glasgow Coma Scale) at hospital arrival as well as GCS and mRS (modified Rankin scale) at discharge. The median length of hospital stay was lower among those who died compared to those alive at 30 days (4.36 vs. 9.56 days) which was statistically significant (p < 0.001) (Table 2).

**Table 2:** Some selected prognostic factors for stroke patients during the 30-day follow-up among adult patients admitted to stroke unit of JUMC from March 10-July 10, 2017.

Some selected prognostic factors		Died at 30-day	Alive at 30-day	χ2	p-value
At hospital arrival	Time interval from onset of stroke to hospital arrival [median(IQR)]	19.0(11.88–75.00)	28.0(8.50–67.50)	3.0	0.558
	NIHSS (Mean ± SD)	21.85 ± 7.18	13.15 ± 6.10	30.61	0.000
	GCS(Mean ± SD)	9.74 ± 3.60	13.09 ± 2.71	40.12	0.000
At hospital discharge	NIHSS (Mean + SD)	15.22 ± 6.70	9.77 ± 5.50	6.36	0.095
	GCS (Mean ± SD)	13.22 ± 2.54	14.52 ± 1.34	9.22	0.010
	mRS(Mean + SD)	5.44 ± 1.16	3.37 ± 1.18	76.69	0.000
	Length of hospital stay[Median(IQR)]	4.36(2.22–6.41)	9.56(5.95–14.17)	27.38	0.000

*GCS: Glasgow coma scale, IQR: Interquartile range, mRS: modified Rankin score, NIHSS: National institute of health stroke scale, SD: Standard deviation.

On multivariable Cox regression analysis, elevated ALT (alanine aminotransferase) level, diagnosis of stroke clinically alone, development of brain edema during hospitalization and NIHSS≥13 during hospital arrival were the independent predictors of 30-day mortality.

The 30-day mortality of patients who had severe to very severe NIHSS (≥13) during hospital arrival was 6.5 times more likely compared to patients with mild to moderate NIHSS (<13) (AHR: 6.49, 95% CI: 1.90–22.22, p = 0.003). Similarly, the 30-day mortality rate of patients who had elevated ALT levels during hospital arrival was about 4 times more likely compared to patients who had normal ALT levels (AHR: 3.77, 95% CI: 1.34–10.57, p = 0.012). Moreover, patients who had stroke diagnosed clinically without imaging confirmation were 4 times more likely to die at 30-day compared to patients who had diagnosed by imaging modalities (AHR: 3.90, 95 CI: 1.49–10.26, p = 0.006). Finally, the 30-day mortality rate of patients who developed brain edema (increased intracranial pressure) during hospitalization was about 4 times more likely compared to patients without brain edema (AHR: 4.28, 95% CI: 1.61–11.37, p = 0.004) (Table 3).

**Table 3:** Predictors of 30-day mortality among adult stroke patients admitted to stroke unit of JUMC.

Variables		Died at 30-day	Alive at 30-day	CHR 95%CI	p-value	AHR 95%CI	p-value
GCS of the patient on hospital arrival	≤8	12	5	9.00 (3.76–21.54)	<0.001		
	9–12	13	20	3.66 (1.56–8.57)	0.003		
	13–15	9	56	1.00			
NIHSS at hospital arrival	≥13	31	41	8.01 (2.45–26.23)	0.001	6.49 (1.90–22.22)	0.003
	<13	3	40	1.00		1.00	
Temperature at hospital arrival	>37.1 °C	10	6	3.03 (1.44–6.36)	0.003		
	≤37.1 °C	24	75	1.00			
Neck stiffness presentation	Yes	8	7	2.32 (1.05–5.18)	0.037		
	No	26	74	1.00			
Comatose presentation	yes	7	4	4.66 (2.02–10.77)	<0.001		
	No	27	77	1.00			
Diagnosis	Clinically	22	32	2.57 (1.27–5.19)	0.009	3.90 (1.49–10.26)	0.006
	Imaging	12	49	1.00		1.00	
Type of the stroke	Hemorrhagic	23	33	2.56 (1.25–5.25)	0.011		
	Ischemic	11	48	1.00			
ALT level	Elevated	7	9	2.60 (1.06–6.37)	0.038	3.77 (1.34–10.57)	0.012
	Normal	15	63	1.00		1.00	
Brain edema complication	Yes	20	15	4.86 (2.44–9.60)	<0.001	4.28 (1.61–11.37)	0.004
	No	14	66	1.00		1.00	
Aspiration pneumonia	Yes	12	11	2.88 (1.42–5.83)	0.003		
	No	22	70	1.00			

*AHR: Adjusted Hazard ratio, ALT: Alanine amino transferase, CHR: Crudes hazard ratio, GCS: Glasgow coma scale, 30D: Within 30 day mortality after admission, NIHSS: national institute of health stroke scale.

**Fig. 1:**
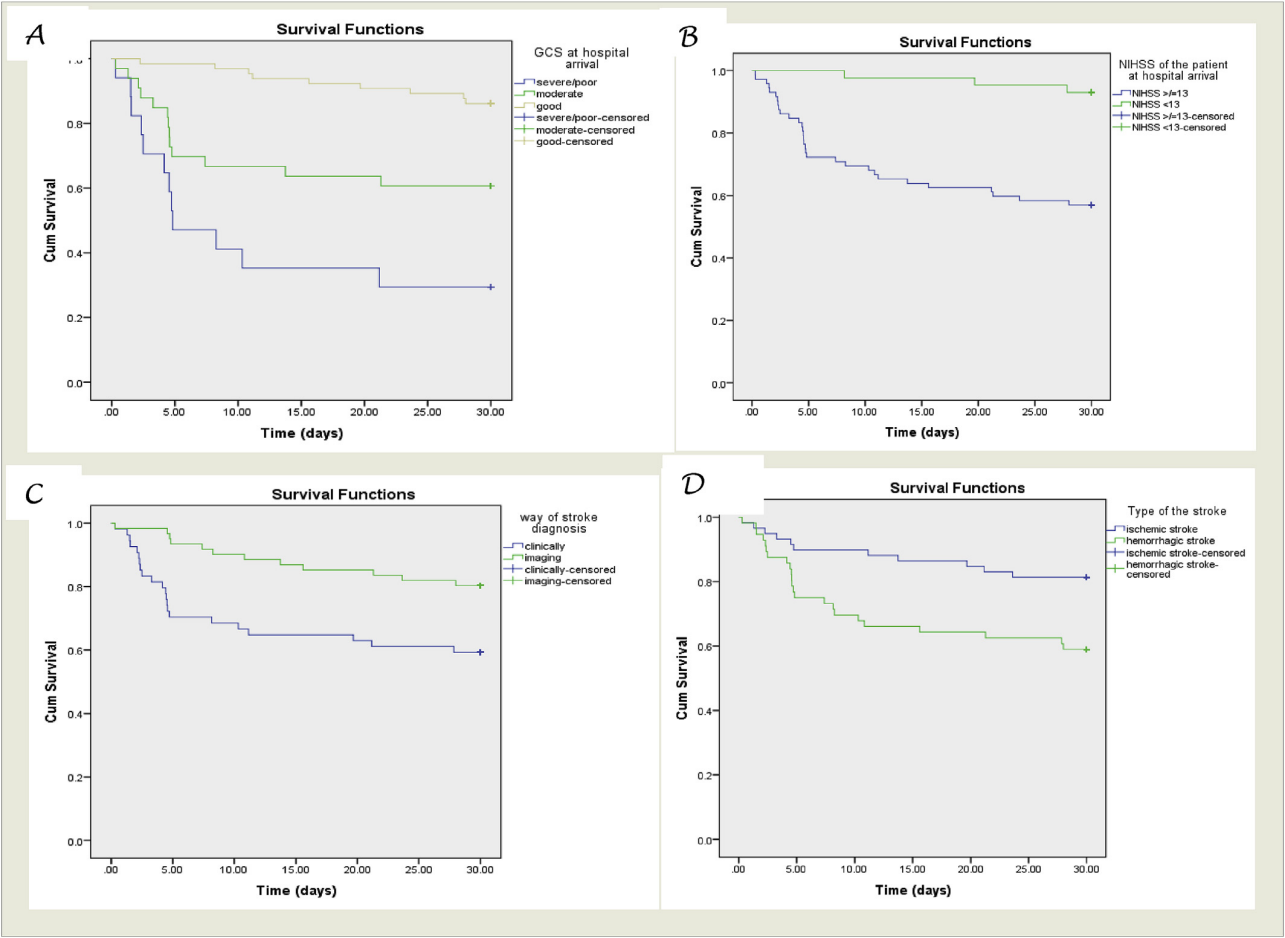
Survival probability curves derived from Log rank Kaplan Meier of mortality after 30 days follow up and GCS at hospital arrival (A), NIHSS of patient during hospital arrival (B), diagnostic modalities (C) and type of stroke (D).

Survival probability curves derived from Log-rank Kaplan Meier of 30-day with different factors were shown (Fig. 1).

### 3.2 60-day baseline characteristics and follow-up outcome

The mean age of patients who died at 60-day was 56.09 ± 14.10 years and those alive at 60-day was 54.28 ± 14.20 years. From the socio-demographic and other baseline characteristics, occupational status had an association with stroke mortality at 60-day upon the chi-square (p = 0.028) (Table 4).

**Table 4:** Sociodemographic and other baseline characteristics and 60-day mortality among adult patients admitted to stroke unit of JUMC from March 10-July 10, 2017.

Variables		Died at 60-day (n = 46)	Alive at 60-day (n = 68)
Age (years)	Mean ± SD	56.09 + 14.10	54.28 ± 14.20
	<45	12(26.1%)	12(17.6%)
	45–65	21(45.7%)	12(17.6%)
	>65	13(28.3%)	14(20.6%)
Sex	Male	29(63.0%)	43(63.2%)
	Female	17(37.0%)	25(36.8%)
Residence	Rural	34(73.9%)	49(79.1%)
	Urban	12(26.1%)	19(27.9%)
Marital status	Married	43(93.5%)	60(88.2%)
	Widow	3(6.5%)	8(11.8%)
	Divorced	–	–
Ethnicity	Oromo	37(80.4%)	43(63.2%)
	Kafa	2(4.3%)	13(19.1%)
	Amhara	2(4.3%)	6(8.8%)
	Dawuro	3(6.5%)	3(4.4%)
	Others	2(4.3%)	3(4.4%)
Religion	Muslim	33(71.7%)	36(52.9%)
	Orthodox	10(21.7%)	25(36.8%)
	Protestant	2(4.3%)	7(10.3%)
	Traditional belief	1(2.2%)	0(0%)
Education status	Unable to read and write	21(45.7%)	20(29.4%)
	Able to read and write, informal education	15(32.6%)	33(48.5%)
	Elementary school (1–8)	8(17.4%)	9(13.2%)
	Secondary school (9–12)	1(2.2%)	2(2.9%)
	College/university or above	1(2.2%)	4(5.9%)
Occupational status (over the last 1years)	Agriculture/farmer	18(39.1%)	25(36.8%)
	Homemaker/housewives	18(39.1%)	22(32.4%)
	Merchant	1(2.2%)	10(14.7%)
	Retired	2(4.3%)	4(5.9%)
	Government employee	1(2.2%)	4(5.9%)
	Other own business work	2(4.3%)	3(4.4%)
	Skilled/unskilled manual labor/daily worker	4(8.7%)	0(0%)
Body mass index (BMI) (kg/m^2^)	Mean ± SD	21.21 ± 3.16	21.25 ± 3.42
	≤18.5 (underweight)	7(15.2%)	16(23.5%)
	18.6–24.9 (normal)	32(69.6%)	42(61.8%)
	25.0–29.9 (overweight)	7(15.2%)	10(14.7%)
Home distance of the patient from hospital (km)	Median (IQR)	45(18.0–68.10)	50.0(13.0–106.55)
	≤10 km	7(15.2%)	16(23.5%)
	10.01–50 km	24(52.2%)	20(29.4%)
	50.01–100 km	8(17.4%)	20(29.4%)
	>100 km	7(15.2%)	12(17.6%)
Living situation during pre-stroke	Independent at home	37(80.4%)	61(89.7%)
	Dependent at home	8(17.4%)	5(7.4%)
	hospital/health center	1(2.2%)	2(2.9%)
Feeding habits	Mixed diet	37(80.4%)	57(83.8%)
	Non vegetarian	6(13.0%)	8(11.8%)
	Vegetarian	3(6.5%)	3(4.4%)
Approximated monthly income (ETB)	Median (IQR)	500(137.50–770)	550(200–1200)
	<500 birr	31(67.4%)	34(50.0%)
	501-1000 birr	8(17.4%)	16(23.5%)
	>1000 birr	7(15.2%)	18(26.5%)

*Ethnicity others: Silte, Yem, Tigire, Nuwer: ETB: Ethiopian birr, SD: standard deviation; IQR: interquartile range.

At 60-day follow-up, 68 (58.6%) patients were alive, 46 (39.7%) were died and 2 (1.7%) were lost to follow-up. All patients that were alive during the end of 60-day were present at their home. The 60-day crude mortality in the current study population was 30 (53.6%) for hemorrhages and 16 (26.2%) for IS which was statistically significant (HR: 2.42, 95% CI: 1.32–4.44, p = 0.004).

The median (IQR) length of survival after hospital admission for patients who died within 60 days was 9.3(3.93–33) days and the median length of survival after hospital discharge was 18.0(12.8–32.2) days. The median (IQR) length of survival after hospital admission for IS and HS patients who died within 60 days was 16.7(3.4–40.2) and 7.8(3.7–29.3) days, respectively. The median (IQR) length of survival after hospital discharge for IS and HS patients died within 60 days after admission was 13.7(9.6–27.5) days and 21.3(14.6–33) days, respectively. There was a significant difference between patients who died and survived to 60-day from hospital admission with respect to NIHSS and GCS at hospital arrival as well as GCS and mRS at discharge. The median length of hospital stay was lower among those who were died compared to alive at 60 days (4.36 vs. 9.56 days) which was significantly different (p < 0.001) (Table 5).

**Table 5:** Some selected prognostic factors of stroke 60- day follow-up among adult patients admitted to stroke unit of JUMC from March 10-July 10, 2017.

Some prognostic factors		Died at 60th day	Alive at 60th day	χ2	p-value
At hospital arrival	Time interval from onset of stroke to hospital arrival[median(IQR)]	19(11.50–65.45)	29(8.25–71.38)	1.15	0.886
	NIHSS (Mean ± SD)	21.1 ± 7.2	12.2 ± 5.4	32.90	0.000
	GCS(Mean ± SD)	10.2 ± 3.6	13.3 ± 2.6	24.30	0.000
At hospital discharge	NIHSS (Mean ± SD)	16 ± 6.5	8.50 ± 4.1	22.76	0.000
	GCS (Mean ± SD)	13 ± 2.4	14.8 ± 0.7	16.61	0.000
	mRS (Mean ± SD)	5.2 ± 1.1	3.18 ± 1.1	55.70	0.000
	Length of hospital stay[median(IQR)]	4.6(2.5–8.7)	9.71(5.7–13.9)	15.13	0.002

*GCS: Glasgow coma scale, IQR: Interquartile range, mRS: Modified Rankin score, NIHSS: National institute of health stroke scale, SD: Standard deviation.

On multivariable Cox regression analysis, discharge against medical advice and mRS at discharge were the independent predictors of 60-day mortality. The 60-day mortality rate of patients who had severe mRS (4–5) during discharge was about 4 times more likely compared to with mild to moderate mRS (≤3) (AHR: 3.64, 95% CI: 1.01–13.16, p = 0.049). Similarly, the 60-day mortality rate of patients who left against medical advice (LAMA) on self and family request from the hospital was 6.5 times more likely compared to patients that were discharged with medical advice (AHR: 6.40, 95% CI: 2.31–17.73, p < 0.001) (Table 6).

**Table 6:** Predictors of 60-day mortality among adult stroke patients admitted to stroke unit of JUMC.

Variables		Died at 60-day	Alive at 60- day	Crude HR 95%CI	p-value	AHR 95%CI	p-value
GCS of the patient on hospital arrival	≤8	14	3	7.78 (3.67–16.48	<0.001		
	9–12	18	15	3.34 (1.67–6.73)	0.001		
	13–15	14	50	1.00			
NIHSS at hospital arrival	≥13	39	33	4.51 (2.02–10.10)	<0.001		
	<13	7	35	1.00			
Coma presentation	Yes	8	3	3.90 (1.81–8.38)	0.001		
	No	38	65	1.00			
Type of the stroke	Hemorrhagic	30	26	2.42 (1.32–4.44)	0.004		
	Ischemic	16	42	1.00			
ALT level	Elevated	10	6	2.80 (1.32–5.91)	0.007	2.72 (0.98–7.56)	0.055
	Normal	22	55	1.00			
Brain edema complication	Yes	22	13	3.29 (1.84–5.90)	<0.001		
	No	14	55	1.00			
Aspiration pneumonia	Yes	14	9	2.41(1.23–4.52)	0.006		
	No	32	59	1.00			
Discharge condition of patient	LAMA	11	5	7.42 (3.13–17.62)	<0.001	6.40 (2.31–17.73)	<0.001*
	With medical advice	10	63	1.00			
mRS at discharge	4–5	17	27	5.09 (1.71–15.16)	0.003	3.64 (1.01–13.16)	0.049*
	≤3	4	41	1.00			
NIHSS at hospital discharge	≥13	13	12	5.09 (1.71–15.16)	0.003		
	<13	8	56	1.00			
GCS of the patient at discharge	≤8	1	0	23.13 (2.64–202.48)	0.005		
	9–12	6	2	5.22 (1.99–13.66)	0.001		
	13–15	14	66	1.00			
Length of hospital stay (days)	>14	5	17	0.23 (0.09–060)	0.003		
	7.01–14	12	32	0.30 (0.51–0.58	<0.001		
	≤7	29	19	1.00			

*AHR: adjusted Hazard ratio, ALT: Alanine amino transferase, GCS: Glasgow coma scale, 60D: Within 60 day mortality after admission, LAMA: Left against medical advice, NIHSS: National institute of health stroke scale, mRS: modified Rankin score.

**Fig. 2:**
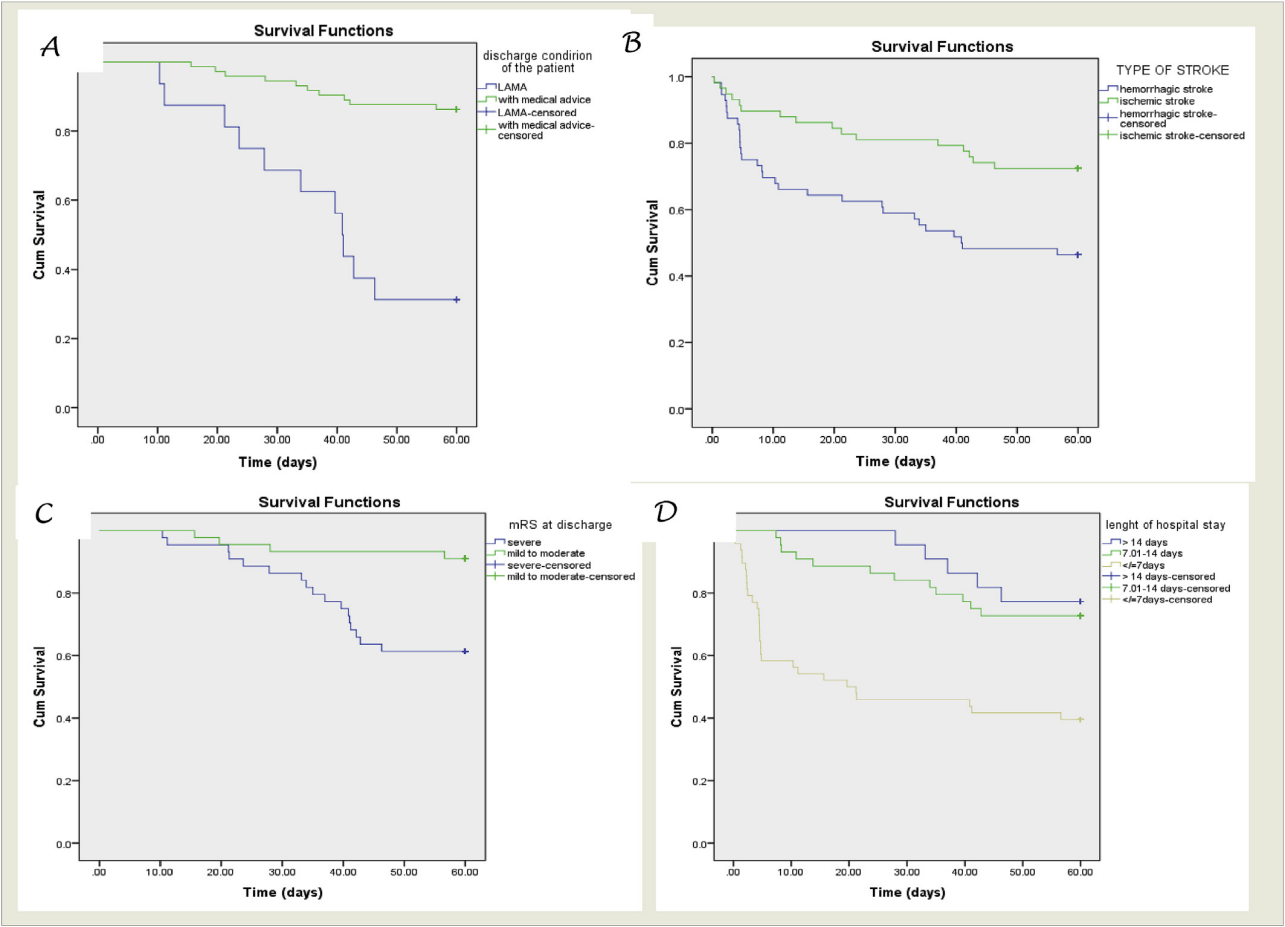
Survival probability curves derived from Log rank Kaplan Meier of mortality after 60 day follow up and type of stroke (A), discharge condition of the patient (B), mRS at discharge (C) and length of hospital stay (D).

Survival probability curves derived from Log-rank Kaplan Meier of 60 days with different factors were shown (Fig. 2).

## 4 Discussion

This prospective cohort study was conducted among stroke patients in Ethiopia to identify the rate and determinants of stroke mortality by following the outcome of the patients after patients were discharged from the hospital. On 30-day follow-up, 34 (29.3%) patients were died which was comparable with the study in Gambia 27% [46] and Uganda 26.8% [11]. This was also similar to the 30-day case fatality rate of 30% reported in a systematic review of hospital-based prospective studies in Sub-Saharan [32]. However, the rate was higher compared to study in Nigeria 17.7% [47] and Cameron 23.2% [48]. The 30-day mortality was lower than a study in Vietnam 36.4% [49] and Mozambique 49.6% [50]. The 30-day crude mortality in the current study population was higher for HS patients, which was statistically significantly similar to previous studies [[48], [49], [50], [51]]. The mortality rate might vary with the severity of the stroke, set up of the hospital, complications, available resources, comorbidities and experts available in caring of the patients.

Elevated ALT level, diagnosis of stroke clinically alone, development of brain edema during hospitalization and NIHSS≥13 during hospital arrival were the independent predictors of 30-day mortality. A study in Vietnam showed that the predictors of 30-day mortality were hemorrhagic stroke type, worse pre-stroke mRS, disturbed consciousness, absence of observed weakness at presentation, higher diastolic blood pressure, higher glucose levels, current tobacco smoking and history of hypercholesterolemia [49]. Another study in Cameron revealed that fever, swallowing difficulties, high NIHSS and elevated systolic blood pressure were independent predictors of 1-month mortality [48]. Also, a study in Uganda showed that initial level of consciousness, stroke severity at admission and fasting blood sugar were independent predictors of mortality at 30-day [11]. Moreover, a study by Kortazar-Zubizarreta et al. reported that higher stroke severity (NIHSS ≥ 14) and potentially modifiable complications confer an increased risk of stroke-related death [8]. A study by Saposnik G et al. also reported that stroke severity and certain processes of care were associated with case fatality at 30-day [52].

The NIHSS is a well-validated tool for assessing initial stroke severity, has previously been shown to be associated with mortality [53]. In the majority of studies, stroke severity was the most predictor of mortality at 30 days. Stroke severity on admission which depends on the level of consciousness was the main clinical predictor of early mortality in many previous studies [54,55].

From current study findings, it was prudent to see that patients who had stroke diagnosed clinically without imaging confirmation were about 4 times more likely to die at 30-day compared to patients who had diagnosed by imaging modalities. This was due to poor confirmation of the type of stroke-related to a shortage of radiologists, the presence of only one CT scan, frequent malfunction of the CT scan and the cost of imaging. Clinical factors were used to identify the patient's stroke subtypes in our set up.

Clinical diagnosis of stroke sub-type is erroneous in some cases, therefore brain imaging is mandatory to confirm the stroke, lesion and rule out other causes. However, systematic brain imaging is impossible for all patients because of the shortage of CT scans and generalized social welfare. Clinical decision rules could be used to determine which patients are more likely to have acute ischemic stroke versus acute intracerebral hemorrhage, although these rules have been found to have a limited predictive capacity [56]. The discrepancy in predictors of mortality at 30 days might be due to sampling size, significance value used, eligibility criteria of the patient, dimensions of factors, set up of the hospital for the management, diagnosis, and care of the patients.

Stroke patients in Ethiopia are often significantly delayed in arriving at the hospital or health care because of long transport distances and delayed referral systems. Other factors included economic problems, lack of family or social support systems and patients’ misconceptions about their symptoms. While we do not currently have the capacity for thrombolysis in JUMC, other supportive care measures are available for stroke victims, including airway management, blood pressure control, antiplatelet therapy, anticoagulation and neurosurgical decompression for increased intracranial pressure. The lack of functional CT scanners to diagnose strokes, the lack of ambulances to transport stroke patients to appropriate health care facilities and poorly organized referral systems are major challenges in stroke care in our setting.

Elevated ALT was another is an independent predictor of stroke mortality at 30-day. This finding correlates with the previous studies by Kim et al. that showed an elevated aminotransferase level is a predictor of ICH [57,58] and a study by Bhatia et al. also identified as elevated ALT level was important indicators of 30-day mortality in patients with first-time IS [59]. The association between high ALT and Cardiovascular diseases (CVD) or mortality is explained by CVD risk factors (mostly due to underlying hepatic inflammation or nonalcoholic fatty liver disease) that are more prevalent in subjects with high ALT [60]. Serum aminotransferase levels are known to be associated with cardiovascular risk factors and showed non-linear associations with mortality [57,[61], [62], [63]].

On a 60-day follow-up, 46 (39.7%) patients were died, which was significantly higher among HS stroke as compared to IS patients. Left against medical advice (LAMA) and mRS at discharge were the independent predictors of 60-day mortality. The patients who were LAMA on self and family request from the hospital died at the rate of 6.5 times more than those patients that were discharged with medical advice. This was because most patients LAMA without any diagnosis or management made the condition worse and favors mortality. Thus, appropriate counseling of the patient who LAMA is mandatory. Additionally, for patients discharged to other wards, intensive coordination from for better management and care of the patient is necessary.

The 60-day mortality rate of patients who had severe mRS (4–5) during discharge was about 4 times more likely compared to with mild to moderate mRS (≤3). A similar factor was responsible for the poor functional outcome among the patients seen in the study by Nakibuuka et al. [11]. According to the study by Rathore et al. high mRS on admission depicting functional disability was one of the predictors of high mortality [20]. The early recovery in activities of daily living among stroke patients in developed countries is generally more favorable [11]. Patients with severe mRS are bedridden, incontinent, who require continuous assistance and constant nursing care and attention. These patients had severe medical and neurological disabilities that impair their functional ability, exposing them to different chronic and infectious diseases [64]. This could expose them to mortality and long term disability. Thus, the management and supportive care of the patients should also consider the functional ability of the patient. This could be achieved by a coordinated effort from health professionals and family/caregiver of the patient. Hence, the high death rates in stroke patients could be reduced by implementing preventive and specific therapeutic strategies.

Stroke mortality may be increasing, but there are no population-based incidence studies that come close to meeting accepted ideal standards [32]. Furthermore, the majority of cases were identified in hospitals and so are unlikely to be representative of the totality of stroke cases in the community [65]. Only a few reliable data are available to identify risk factors for stroke in most LMICs regions and particularly for HS [7]. Although the burden of stroke has increased in developing countries, health care services have not caught up due to scant resources for acute care and rehabilitation [10,30]. The challenges to providing health care services for stroke in developing countries include lack of awareness about stroke risk factors, economic resources for well-functioning healthcare systems, unaffordable cost of tPA (tissue-type plasminogen activator), lack of rehabilitation facilities and preference for alternative and complementary medicines over modern medicines [12,28,30].

One other potential reason for these poorer outcomes may be uncertainty among physicians about how best to manage patients presenting with acute stroke when CT is unavailable to distinguish IS from HS [66]. The number of stroke patients receiving r-tPA in the LMICS is extremely low. Prehospital delay, financial constraints and lack of infrastructure are the main barriers of thrombolytic therapy [42,44,67]. Furthermore, as most guidelines are based on data from developed countries, uncertainty remains regarding the best management of the unknown types of stroke in LMICs [1]. Like other LMICs resources for stroke care and rehabilitation are deficient in Ethiopia [17].

Stroke is a heterogeneous syndrome and providing appropriate management to patients with acute stroke depends on the underlying etiology of the stroke [66,68]. Time to care is also very important to save brain tissue but there are unique challenges in LMICs [34]. Identification of early outcomes, post-stroke mortality and their predictors are important in stroke management strategies [11]. To reduce the overall burden of stroke in the society organized approach is needed to predict mortality and morbidity in stroke especially aggressive management for complications of stroke [22,44].

### 4.1 Strengths and limitations of the study

An important strength of our study was its prospective study design and the enrollment of consecutive patients. All data were collected prospectively and uniformly which was based on a general neurology clinical practice setting. We scanned every factor longitudinally as far as possible within our infrastructure, then conclude by a standard statistical method. The study provides a preliminary database on mortality which can inform stroke management strategies and interventions required to decrease mortality associated with stroke. We have performed a detailed initial assessment including NIHSS, GCS, and mRS to evaluate for determinants of outcome in a series of patients. Additionally, only a few patients were lost to follow-up. Also, we have used methods of survival analysis with competing for risk that allowed us to estimate the risks of mortality at 30 and 60-day.

There were some limitations to our study that deserve comment. First, our study recruited patients who were present in the hospital for stroke were introducing a selection bias whereby stroke severity extremes might not be included (very minor or rapidly fatal). Our study was based on data acquired in a single hospital, may not be readily comparable to the patterns of in‐hospital stroke mortality in multicenter studies. However, we had attempted to include all consecutive patients with acute stroke to reduce any selection bias. Although our overall findings were consistent, hospital-based controls could underestimate the true association for some risk factors.

Secondly, the sample size was small hampering the analysis of some prognostic indicators due to the short recruitment period. Sixty-day follow-up is too short to identify the outcome of acute stroke. Indeed, a prospective community-based cohort design would require thousands of stroke-free subjects who would need to be followed up for several years to know the outcome of patients even after patients were discharged. In the LMIC setting, resources are not available for this and results are urgently required to help implement the stroke intervention like patient management, prevention, acute care, and rehabilitation services.

Third, we followed up patients by telephone, not by face to face interview. Thus, the detailed data of stroke severity, recovery and disability could not be collected in this study and the accuracy of these self-reported events needs to be evaluated. Finally, diagnostic investigations were undertaken based on the subject's syndrome, rather than a complete evaluation of the cases to rule out as evidenced by the similar proportions of inadequate workup. In many cases, the investigators were not the primary treating physicians and it was difficult to validate some of the diagnosis made by other physicians. Despite these limitations, this study provides novel information on similarities between variables associated with case fatality at different points.

## 5 Conclusions

The increasing burden of chronic diseases including stroke in LMICs like Ethiopia poses a challenge to the health care system and the community as a whole. Stroke has significantly affected the morbidity and mortality in our country. At 30-day follow-up, the majority of them were alive, but about one-third of the patients were died. Elevated ALT level, diagnosis of stroke clinically alone, development of brain edema during hospitalization, and having NIHSS≥13 during hospital arrival were the independent predictors of 30-day mortality.

At 60-day follow-up, the majority of them were alive, about two-fifths of the patients were died. LAMA on discharge and mRS at discharge were the independent predictors of 60-day mortality. In general, the mortality rates of stroke in JUMC was similar to other most LMICs. The disparity in mortality of stroke across different regions of the world was likely to be due to a combination of differences in risk factor prevalence, environmental, facility for the care, genetic and study design.

Therefore, urgent strategic intervention is needed to overcome the current factors associated with mortality among stroke patients in LMICs including Ethiopia. From a public health point of view, preventive measures to reduce the risk of stroke would provide additional cross-cutting benefits. Efforts should be made to establish best practices for acute stroke care in our settings. Educational programs for front-line health-care providers and focusing on simple supportive interventions could improve outcomes in settings where advanced diagnostics and treatment of stroke remain limited. The Ethiopian ministry of health should develop and implement generalized protocol guidelines for in-hospital management and post-stroke follow-up. Different organizations that work in areas of non-communicable disease should focus on the current debilitating conditions of stroke in SSA including Ethiopia through better funding of the health care system to improve the quality of care. In the absence of these interventions, stroke-related mortality will regrettably continue. Development of a network of local and regional stroke centers with expertise in early stroke evaluation and management may address some of the challenges around timely diagnosis and referral.

Finally, there is a need to provide better care and future planning for stroke patients as an emergency diagnosis and treatment to minimize mortality and disability among stroke survivors. A prospective community-based stroke incidence and prevalence studies are required to define the true mortality of stroke. Hence, future studies collecting data from a large number of centers, and carrying out a pooled data analysis may detect further determinants of stroke mortality.

## Ethical approval

Ethical clearance was obtained from the Institutional Review Board (IRB) of Jimma University, Institute of health. With reference number of IHRPGC/107/207.

## Sources of funding

This work was funded by Jimma University. The funding body did not have any role in study design, data collection, data analysis, interpretation of data or in writing the manuscript.

## Author contribution

GF contributes to the design of the study, analysis, interpretation and writes up of the manuscript. AK made the data analysis and interpretation of the data. LC, TM, and ET contributed to the design of the study, drafting and edition of the manuscript. All authors critically revised the manuscript and have approved the final manuscript.

## Registration of research studies

1. Name of the registry: RESEARCH REGISTRY, <u>https://www.researchregistry.com</u>
2. Unique Identifying number or registration ID: researchregistry5260
3. Hyperlink to the registration (must be publicly accessible): <u>https://www.researchregistry.com/register-now#home/registrationdetails/5de2256c9d9d030015507006/</u>

## Guarantor

Ginenus Fekadu.

## Consent

Not applicable. No individual person's personal details, images or videos are being used in this study.

## Availability of data and materials

The datasets used and/or analyzed during the current study are available from the corresponding author on reasonable request.

## Provenance and peer review

Not commissioned externally peer reviewed.

## Declaration of competing interest

The authors declared that they have no competing interest.

## Abbreviations

AHR: Adjusted hazard ratio
AOR: Adjusted odds ratio
CVA: Cerebrovascular accident
CVDs: Cardiovascular diseases
GBD: Global Burden of Diseases
GCS: Glasgow coma scale
HS: Hemorrhagic stroke
HTN: Hypertension
ICH: Intracerebral hemorrhage
IS: Ischemic stroke
JUMC: Jimma university medical center
LAMA: left against medical advice
LMICs: Low and middle income countries
mRS: Modified rankling score
NIHSS: National Institute of Health Stroke Scale
SSA: Sub Saharan Africa
SU: Stroke unit
WHO: World Health Organization
